# Living in Promiscuity: The Multiple Partners of Alpha-Synuclein at the Synapse in Physiology and Pathology

**DOI:** 10.3390/ijms20010141

**Published:** 2019-01-02

**Authors:** Francesca Longhena, Gaia Faustini, Maria Grazia Spillantini, Arianna Bellucci

**Affiliations:** 1 Division of Pharmacology, Department of molecular and Translational Medicine, University of Brescia, Viale Europa 11, 25123, Brescia, Italy; f.longhena@unibs.it (F.L.); g.faustini004@unibs.it (G.F.); 2 Department of Clinical Neurosciences, Clifford Allbutt Building, University of Cambridge, Cambridge CB2 0AH, UK; mgs11@cam.ac.uk; 3 Laboratory for Preventive and Personalized Medicine, Department of molecular and Translational Medicine, University of Brescia, Viale Europa 11, 25123, Brescia, Italy

**Keywords:** α-synuclein, synaptic proteins, conformational plasticity, synucleinopathies, interactome

## Abstract

Alpha-synuclein (α-syn) is a small protein that, in neurons, localizes predominantly to presynaptic terminals. Due to elevated conformational plasticity, which can be affected by environmental factors, in addition to undergoing disorder-to-order transition upon interaction with different interactants, α-syn is counted among the intrinsically disordered proteins (IDPs) family. As with many other IDPs, α-syn is considered a hub protein. This function is particularly relevant at synaptic sites, where α-syn is abundant and interacts with many partners, such as monoamine transporters, cytoskeletal components, lipid membranes, chaperones and synaptic vesicles (SV)-associated proteins. These protein–protein and protein–lipid membrane interactions are crucial for synaptic functional homeostasis, and alterations in α-syn can cause disruption of this complex network, and thus a failure of the synaptic machinery. Alterations of the synaptic environment or post-translational modification of α-syn can induce its misfolding, resulting in the formation of oligomers or fibrillary aggregates. These α-syn species are thought to play a pathological role in neurodegenerative disorders with α-syn deposits such as Parkinson’s disease (PD), dementia with Lewy bodies (DLB), and multiple system atrophy (MSA), which are referred to as synucleinopathies. Here, we aim at revising the complex and promiscuous role of α-syn at synaptic terminals in order to decipher whether α-syn molecular interactants may influence its conformational state, contributing to its aggregation, or whether they are just affected by it.

## 1. Introduction

Alpha-synuclein (α-syn) is a small protein belonging to the synuclein superfamily that also encompasses β-synuclein (β-syn) and γ-synuclein (γ-syn). These are evolutionary conserved proteins with distinctive functions that share sequence homology with other proteins, such as the class A2 lipid-binding domains of the apolipoproteins, 14-3-3 chaperones and several small heat-shock-proteins, but whose ancestor remains unknown [1]. Alpha-synuclein is the most frequently observed synuclein across all vertebrate organisms, supporting the notion that it regulates some essential physiological functions [1]. Alpha-synuclein is abundant in neurons of the nervous system, where it localizes in presynaptic terminals [2,3,4] and modulates synaptic functions [5,6,7]. This notwithstanding, α-syn is among the last presynaptic proteins to become enriched at the synapse [8] and it does not seem to be involved in synaptic development [9]. Over the last few years, experimental evidence has indicated that mitochondria, endoplasmic reticulum (ER) and nuclei also contain α-syn [2,10,11,12,13,14,15,16,17,18,19,20], although at lower levels than those observed at synaptic sites [8,21,22,23]. While it is clear that α-syn can modulate synaptic activity, the function and presence of the protein within these organelles is still a matter of debate. Similarly, the significance of its modulatory action on ER-Golgi transport and cytoskeletal organization is still discussed [24,25,26,27,28,29,30,31,32,33]. Interestingly, it has also been found that α-syn can contribute to tumorigenesis [34] and is expressed in a variety of cancers including tumors with neuronal differentiation, melanomas and meningiomas [35,36,37]. Although the wide subcellular distribution of α-syn in neurons may not necessarily reflect some relevant functions, it is indicative of its remarkable conformational plasticity [38]. As a natively unfolded protein with intrinsically disordered profile [39], α-syn can easily shift its structure and interactions, which can be significantly influenced by the surroundings, i.e., neighboring proteins, lipid membranes, redox state, local pH [40,41,42,43,44,45,46,47,48,49,50], and these characteristics place α-syn among the intrinsically disordered proteins (IDPs). Notably, recent findings support that high-affinity and dynamic complex formation between two oppositely charged IDPs is possible without the formation of a sequence-specific structure or the need for folded domains [51]. As a consequence, the binding selectivity of charged IDPs, such as α-syn, may be established through the influence of regulatory mechanisms that may result from its subcellular localization, or synchronized expression during relevant stages of development or of cell cycle [52]. In light of its elevated molecular plasticity, α-syn may also behave like a hub within protein interaction networks [38], with its conformational state in the different subcellular sites (unfolded/structured), governing its interacting abilities and influencing the interconnected partners as a consequence. Contrariwise, it is foreseeable that α-syn may be affected by the molecular features of its partners that might impinge on its conformation and function with variable modality and relevance. While these considerations are central for a proper understanding of α-syn physiological functions, they become even more meaningful when considering that this protein plays a pathogenic role in a series of neurodegenerative disorders, collectively defined as synucleinopathies [53,54,55]. In the late 1990s, α-syn and its fibrils were first described as the main protein constituent of Lewy bodies (LB) and Lewy neurites (LN), the intraneuronal and intraneuritic insoluble protein deposits that characterize the brain of patients affected by Parkinson’s disease (PD) and dementia with LB (DLB), as well as of the glial cytoplasmic inclusions (GCI) that are typically found in multiple system atrophy (MSA) brains [54,55]. Later studies have shown that α-syn is also present in the brain of patients affected by Alzheimer’s disease (AD), especially the LB variant of this disorder, and in LB dysphagia [53,56,57]. In the last 20 years, research findings have demonstrated that mutations or multiplications of the α-syn gene (SNCA) correlate with the onset of PD or DLB [58]. Moreover, a staging scheme for PD has been proposed, based on the fact that the stereotyped pattern of the gradual caudo-rostral diffusion of LB, within interconnected brain regions, correlates with symptoms progression in patients [59,60]. This evidence not only substantiated the centrality of insoluble α-syn deposition in the pathophysiology of PD, but became central to the prion hypothesis. According to this theory, some α-syn seeds of aggregation are transmitted from one neuron to another, in this way propagating α-syn pathological misfolding in a prion-like fashion [61,62,63]. Indeed, a plethora of experimental evidence has confirmed that α-syn can be transmitted from cell-to-cell, exploiting various mechanisms [64,65]. Factual evidence of the prion-like behavior of α-syn was supported by the finding that striatal grafts in transplanted PD patients develop LB pathology [66,67] and that MSA or PD patient-derived brain homogenates could induce pathological α-syn deposition when injected in recipient cells, or in the brain of mice and monkeys [68,69]. Remarkably, injection of synthetic α-syn fibrils in different areas of the brain was also found to initiate trans-synaptic spreading of pathology within interconnected brain regions, causing PD-like degeneration, acting as a seed for endogenous α-syn conformational shift toward insoluble species [70,71,72,73,74]. However, it appears that the diverse characteristics of the intracellular milieu of neurons and oligodendrocytes differentially contributes to imprinting the self-propagating conformation of the pathological α-syn strains, with oligodendrocyte-derived GCI-like strains maintaining their seeding ability even when propagated in neuronal cells [74]. All the events described above can severely influence the α-syn functional spectrum, and in particular, its synaptic actions. Indeed, α-syn synaptic interactome/functions seem to be strictly dependent on the distinct structural conformation adopted by the protein. In view, thatsynaptopathy is emerging as the major trigger for the retrograde neurodegeneration pattern of synucleinopathies [75,76,77], having a more detailed knowledge of α-syn synaptic partners, on the regulatory features of their interaction, on how they may be perturbed by the presence of pathological α-syn aggregates, and on whether and how they may contribute to α-syn deposition and spreading, becomes compelling. A multiple spectrum of synaptic proteins has been found to be modulated by or to affect α-syn directly or indirectly and/or are altered in the brain of PD, DLB or MSA patients and in different experimental models of these disorders [78,79,80,81,82,83,84]. Some of these synaptic partners can either simply serve as adjuvant of normal α-syn function or are influenced by a chaperone-like action of α-syn. Others are clearly emerging as key participants in α-syn aggregation and synaptopathy generation. This review aims to present an integrated view of the results of studies describing the intermodulation of α-syn conformational variants and synaptic proteins in physiology and pathology in the attempt to improve our understanding of the biological basis of synucleinopathies.

## 2. Alpha-Synuclein Structure and Conformational Variety

**Figure 1:**
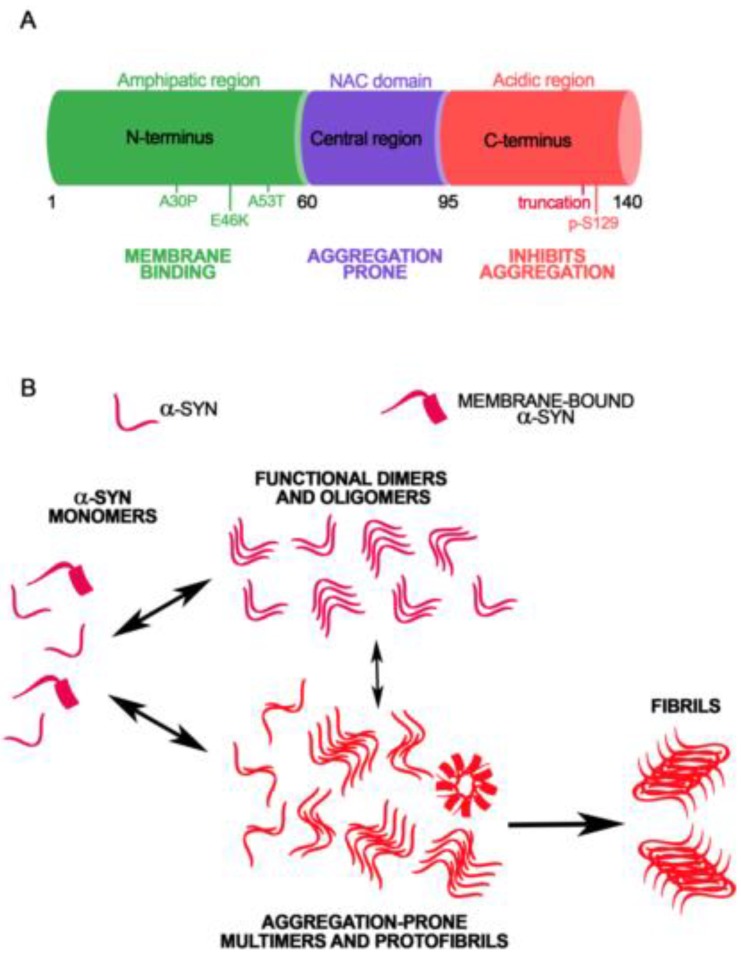
Amino acid sequence and conformational variability of α-synuclein (**A**). The primary amino acid sequence of α-syn can be divided in three main regions: the N-terminal amphipatic domain, the central part containing the NAC sequence and the C-terminal acidic tail. Pathogenic mutations related to familiar forms of PD and the S129 phosphorylation site are also represented (**B**). The elevated structural plasticity of α-syn can give rise to the formation of functional dimers, tetramers and oligomers or high molecular weight aggregation-prone oligomers, protofibrils and fibrils.

Alpha-synuclein is a conserved presynaptic protein counted among the family of IDPs, as it lacks rigid well-defined structure [85,86]. Its conformational plasticity, which depends on its primary amino acid sequence, reflects the ability of α-syn to interact with multiple ligands, including proteins and lipids, and to exert chaperone-like functions [87,88]. The primary sequence of α-syn can be divided into three main regions that, by playing diverse roles in modulating its folding and aggregation state, can differentially affect its interacting capabilities (Figure 1A). The N-terminal domain (aa 1–60), encompassing four highly conserved 11-mer repeats with a KTKGEV consensus sequence involved in the formation of amphipathic α-helices, is essential for membrane binding [89,90,91]. These helices are stabilized by interaction with high-curvature membranes enriched in phospholipids, similarly to synaptic vesicles (SV) [92,93]. This part of the protein can also form α-helical oligomers following acetylation, suggesting that this post-translational modification impact on the structural and functional properties of α-syn [94,95]. Nitration of Tyr39 can disrupt the ability of α-syn to interact with lipid membranes and is also essential for the formation of high-ordered oligomers through 3-nitrotyrosine crosslinking [96,97,98]. The N-terminal portion of the protein includes the sites of three familial PD mutations: A30P, A53T and E46K that can differentially affect the affinity of the protein for lipid membranes. The A53T mutant has comparable affinity for biological membranes relative to wild type (wt) α-syn, while A30P mutation reduces the affinity of the protein for biological membranes and the E46K mutation increases the α-syn membrane [91,99,100,101,102,103]. The central region of α-syn (aa 61–95) comprises the hydrophobic non-amyloid component (NAC) domain: a sequence that is highly aggregation-prone [104] and results necessary and sufficient for α-syn filament formation [105,106]. When α-syn is in a disordered state, this region is shielded from the cytoplasm via transient intramolecular interactions, in order to prevent aggregation [107]. The C-terminal domain of α-syn (aa 96–140) is highly enriched in negatively charged amino acids and proline residues, which are known to disrupt secondary protein structure [108,109]. This region seems to interact with the N-terminal region of α-syn in order to protect the NAC residues, resulting relevant to form compact aggregation-resistant monomeric structures [43,110]. Post-translational modifications of the C-terminal domain can enhance α-syn aggregation propensity and affect its molecular interactions. Phosporylation at Ser129 or nitration at Tyr125, Tyr133 and Tyr136 has been reported to promote the formation of α-syn fibrils or oligomers [97,111,112], alter its conformational state and reduce its membrane-binding affinity [96]. Nonetheless, some studies debate the influence of phosphorylation at Ser129 for α-syn aggregation [113]. Moreover, C-terminally truncated forms of α-syn aggregate faster than full length protein [114,115].

In physiological conditions, α-syn is thought to be natively unfolded, but is slightly more compact than a random coil [41]. During subcellular fractionation steps, the protein is mainly found in the synaptic fractions in association with SV membranes [116]. Depending on the purification and separation conditions, α-syn can be visualized on acrylamide gels at 14–19 kDa and also at 57–58 kDa [117]. A possible explanation of the presence of these high molecular species is that α-syn may also exist natively as a stable tetramer that has been visualized by nuclear magnetic resonance (NMR), analytical centrifugation and scanning transmission electron microscopy (TEM) [118,119,120]. Although the existence of these tetrameric species is debated [74,121,122], they have been found to display an α-helical conformation, and are resultingly resistant to aggregation [119,123]. Rapid changes of environment were found to induce the formation of folding intermediates or kinetically trapped transition states [124]. Moreover, single-particle electron microscopy of purified α-syn revealed the presence of trimeric and dimeric complexes [105,125,126,127,128]. Indeed, purification of α-syn from neuronal and non-neuronal cells resulted in the isolation of different multimeric forms [129], which were easily disassembled during the fractionation or purification steps [130]. Live-imaging experiments on intact neurons have shown that α-syn is able to adopt different conformations depending on its subcellular location or synaptic activity [131,132]. These findings support that each of these α-syn multimers could be involved in specific functions of the protein. The conformational plasticity of α-syn, its elevated concentration at the synapse, and the large number of processes in which it is involved, could contribute on α-syn inducing the formation of high molecular weight species such as fibrils or protofibrils (Figure 1B) [40,133,134,135,136,137,138]. First, during these stochastic events of self-association, α-syn assemblies can be converted into aggregation-competent oligomers (Figure 1B) [133,139] that act as nucleation sites for unfolded monomer, leading to different fibrillar species [40,135,140]. Distinct strains of α-syn fibrils can indeed be obtained by isolation of insoluble α-syn from the brain of patients affected by synucleinopathies [68,141]. Notably, this complexity is partially reproducible also in in vitro conditions, and different experimental approaches can be used to study the dynamics of fibril formation [142], or the inner structure of the human-derived or artificially pre-formed fibrils [143,144,145]. The impact of α-syn mutations on the fibrillation rate of the protein has been investigated using biophysical methods including circular dichroism and differential scanning colorimetry, which, in line with other reports [146,147,148], confirmed that A30P, A53T, E46K mutants exhibit higher aggregation propensity when compared to wt α-syn [144,149,150]. Other biophysical methods, such as Raman Spectroscopy, that can be applied to the study of protein structure, were found to detect slight differences between fibrils obtained from different PD-related α-syn genetic mutations [144]. More recently, Kumar and coauthors [151] developed a method for the identification of amyloid fibrils using chiroptical effects in plasmonic nanoparticles. In particular, they probed the formation of amyloid fibrils based on α-syn, using gold nanorods that bore no apparent interaction with monomeric proteins, but were effectively absorbed onto fibril structures via noncovalent interactions. The amyloid structure could drive a helical nanorod arrangement, resulting in intense optical activity at the surface plasmon resonance (SPR) wavelengths. This technique allowed the detection of protein fibrils with disease relation identified through chiral signals from gold nanorods in the visible and near IR in human brain homogenates of patients affected by PD, although healthy brain samples did not show meaningful optical activity. Finally, NMR spectroscopy and TEM were also successfully applied to the study of α-syn fibrils structure. In particular, they were able to identify a recurrent Greek-key like domain in α-syn fibrils [143,152]. This notwithstanding, one has to be aware that the cellular behavior of IDPs such as α-syn, when evaluated by analytical methods such as NMR, may differ significantly from that observed in the test tube [153]. Therefore, we need to sharpen and improve our technological tools to achieve a complete and reliable understanding of the effective molecular structure of fibrils or oligomers. Since different α-syn strains were also found in the cerebrospinal fluids [154], the development of immuno-based techniques allowing the detection of conformation-specific α-syn particles, might help to elucidate this conundrum and likely also support the diagnosis of synucleinopathies. Similarly, studies addressing how the different α-syn protein partners may impact its conformation may be of invaluable help for establishing whether some of them can enhance α-syn aggregation.

## 3. Alpha-Synuclein Modulation of Protein Trafficking at the Synapse

**Figure 2:**
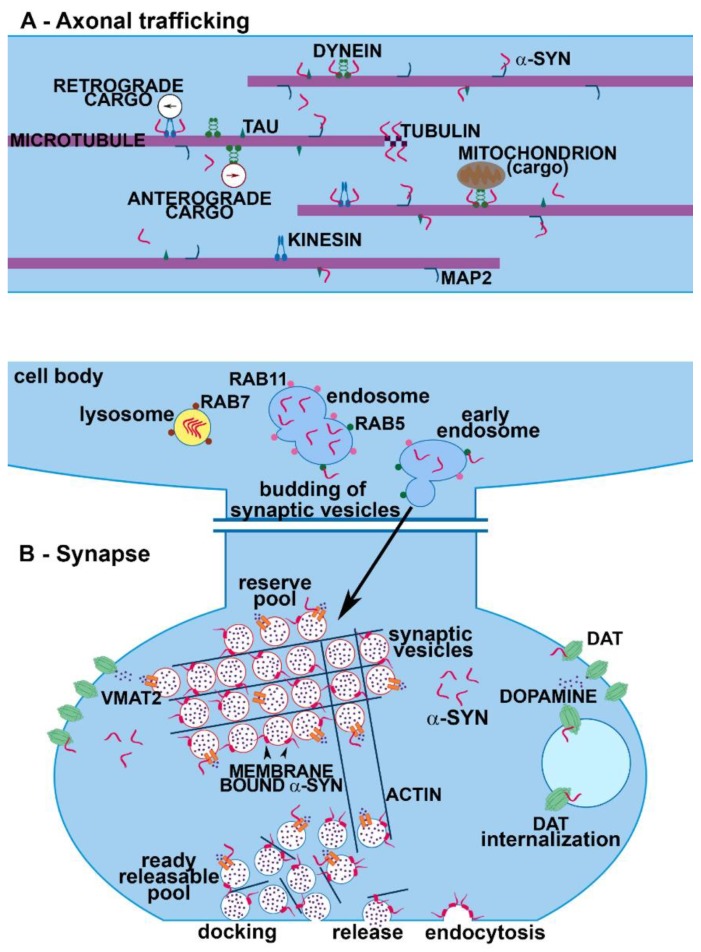
Role of α-syn in axonal trafficking and synaptic terminals. The image is representative of α-syn interactome in axons (**A**) and synapses (**B**). (**A**) Alpha-synuclein interacts with different motor proteins mediating axonal transport, such as kinesins and dyneins, as well as with microtubules, where it contributes to the polymerization of the single tubulin molecules. (**B**) At the synapse α-syn plays multiple important roles in SVs trafficking and refilling and in neurotransmitter release and reuptake. Alpha-synuclein aggregation perturbs the distribution and function of its synaptic partners.

Alpha-synuclein aggregation and the onset of PD are frequently associated with axonal transport defects that strongly depend on microtubule network impairment [155,156]. This event can induce derangement of synaptic terminals and initiate neurodegeneration. As observed by proteomics assays, α-syn interacts with a variety of cytoskeletal proteins contributing to the maintenance of cell structure and protein trafficking [24,26,29,157]. In neurons, microtubules and the associated proteins are fundamental for SV transport. Interestingly, α-syn aggregation has been found to affect microtubule stability in multiple ways. Alpha-synuclein oligomers were found to disrupt axonal integrity in induced pluripotent stem cell (iPSC)-derived human neurons, as they perturbed the correct association of α-syn with kinesin, which is essential for axonal transport [33]. Proteins that govern neuronal trafficking, like kinesin and dynein, which are implicated in the anterograde and the retrograde transport, have been shown to be altered in PD models with a strong association with motor deficits [158]. Dynein co-localizes with α-syn and dynein-dependent axonal transport is severely affected in the absence of α-syn [159] (Figure 2A). In physiological conditions, co-immunoprecipitation experiments confirmed a direct interaction of α-syn with the α and β subunits of tubulin that can promote the polymerization of microtubules. In particular, α-syn endorses microtubule nucleation and enhances the growth rate of neurons [26] (Figure 2A). Moreover, tubulin itself was found to be enriched in LB and seems to potentiate α-syn fibrillization [26]. Alpha-synuclein genetic mutations influence microtubule aggregation and disorganization [160,161] and in PD the protein can act as a microtubule-associated protein that directly or indirectly causes microtubule destabilization and affects its dynamics [162]. Alpha-synuclein monomers are able to bind a plethora of proteins required for anterograde axonal transport, such as Kinesin Family Member 5A (KIF5A), tubulin, microtubule-associated protein 2 (MAP2), and tau [157]. Remarkably, α-syn was reported to directly bind tau in the microtubule-binding domain, thus contributing to the destabilization of microtubules [163] and altering their polymerization [164]. In fact, α-syn has been reported to be able to promote tau oligomerization through a binding or phosphorylation mechanism [163,165,166]. Moreover, it has been shown in vitro that α-syn mutations prompting fibrillization can boost tau assembly by synergistically enhancing the reciprocal fibrillization of tau and α-syn [167]. Since tau binds and stabilizes microtubules, the interaction of α-syn with tau may compromise the integrity of microtubule network, causing axonal dysfunction and neuronal death when this protein aggregates. Microtubule stability is regulated by both phosphorylated and unphosphorylated forms of tau that interact with 14-3-3 proteins including Leonardo (Leo) and D14-3-3ε (Eps) and full-length 14-3-3ζ [168,169,170,171,172,173]. These are cytoplasmic protein chaperones affecting protein folding, trafficking, cytoskeletal reorganization and neurite development by phosphorylating their targets [174]. Alpha-synuclein was also reported to interact with 14-3-3 proteins such as 14-3-3 η [175] and to affect their ligands, such as Protein kinase C (PKC). In particular, α-syn overexpression inhibits the activity of PKC with a toxic mechanism [176]. Interestingly, 14-3-3 proteins were identified among the components of LB of PD patients [177] and can preferentially interact with small oligomeric forms of α-syn [175]. In vitro and in vivo experiments on PD models revealed that the overexpression of some isoforms of 14-3-3 proteins exerts a neuroprotective effect by reducing α-syn inclusion formation [178]. On the other hand, dopamine (DA)-dependent neurotoxicity seems to be mediated by 54-83 kD soluble protein complexes containing α-syn and 14-3-3 protein, which are selectively elevated in the substantia nigra of PD patients [179]. Alpha-synuclein overexpression reduces the levels of 14-3-3, although α-syn knock down does not exert the opposite effect, thus supporting that regulation of 14-3-3 expression is not a function of endogenous α-syn at baseline [180]. The C-terminal phosphorylated form of α-syn specifically interacts with 14-3-3 proteins and modulates cytoskeletal and vesicular protein trafficking [181]. Moreover, 14-3-3 acts as a α-syn chaperone and reduces its seeding potential, uptake and toxicity [175,182]. Another relevant α-syn interacting protein regulating the transport of vesicles along the axons and neurite outgrowth is actin. Alpha-synuclein has been reported to bind to actin [183,184] that has been found to be altered in PD models [185,186]. Changes in α-syn can modulate actin remodeling and dynamics, increasing the plasticity of the cytoskeleton at the synapse [184]. The alteration of actin dynamics is also mediated by interaction of α-syn with spectrin, which causes a mislocalization of a fission protein with a consequent mitochondrial dysfunction, as observed in PD and DLB [187]. At the presynaptic site, the cytoskeletal matrix is composed of a large number of proteins; among them, piccolo and bassoon are involved in the formation of the active zone, where they interact with a variety of proteins involved in its organization. Specifically, piccolo participates in the trafficking of SV at the active zone through a dynamic assembly of actin cytoskeleton [188,189], and bassoon regulates the retrograde axonal transport [190] without directly affecting neurotransmitter release [191]. Although α-syn is important for the regulation of the size of SV and distribution at the active zone [192], there is no reported evidence supporting that it can interact with piccolo and bassoon. However, α-syn overexpression has been found to induce a decrease of piccolo in hippocampal neurons [193], supporting the notion that it may indirectly affect this protein. The intracellular trafficking of organelles, such as vesicles, mitochondria or ER, among cellular compartments is strictly regulated by a family of GTPases called Rab proteins (Rabs) from the Ras super family proteins. They exert a significant role in trafficking, fusion and tethering of membranes of SV or organelles. Alpha-synuclein monomers modulate the internalization of SV through the endocytic pathway, while the interaction with Rab4A plays an important role for protein sorting and for their transport. Moreover, α-syn is sorted to the early endosome with a mechanism dependent on Rab5A and to the late endosome with a mechanism dependent on Rab7 [194] (Figure 2B). On the other hand, Rab11 interacts with endogenous α-syn in vivo and modulates α-syn secretion [195].

## 4. Alpha-Synuclein: A Handyman in SV Machinery

**Figure 3:**
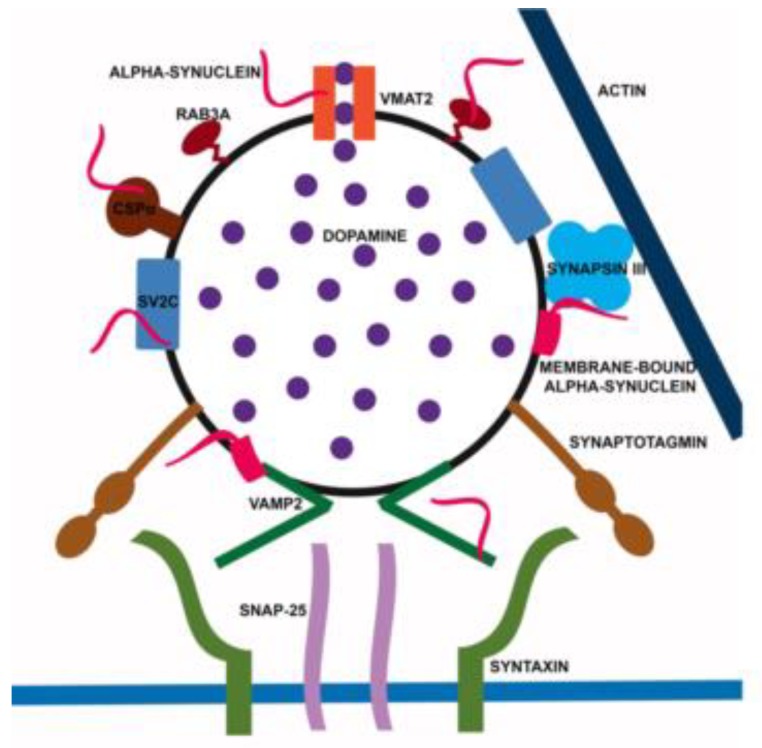
Overview of the α-syn interactome at SV. Alpha-synuclein interacts with multiple SV proteins as well as with SV membranes. By orchestrating its SV partners, α-syn contributes to neurotransmitter filling, SV tethering, docking and fusion. Please note that syn III is the abbreviation for synapsin III.

Alpha-synuclein has represented one of the best markers for presynaptic terminals since it was first identified in association with SV [2]. Nonetheless, α-syn can be considered to be the handyman of the synapse. Indeed, it interacts and cooperates with a high number of proteins and regulates the trafficking of SV [7,9,196]. First, by interacting preferentially with small vesicles [92,93], α-syn is thought to regulate the mobility of SV between the recycling and the resting pools [197] (Figure 2B). The binding of α-syn to SV is Ca^2+^-dependent and involves the aminoacidic portions 1–25 and 65–97 [48,198]. The absence of α-syn causes a depletion of the resting pools, thus blocking the refill of docked SV [199], whereas the overexpression of α-syn inhibits neurotransmitter release by altering the size of the resting pools, likely because a large amount of the protein could perturb SV trafficking to the active zone. Furthermore, aggregated forms of α-syn block SV docking [200,201], suggesting that the protein is involved in multiple steps of SV mobilization. On the SV surface, α-syn interacts with other synaptic proteins, such as a family of phosphoprotein called synapsins [202]. A modest increase of α-syn in the range predicted for gene multiplication reduced the size of SV recycling pools and was associated with reduction of synapsin I and II, complexins and mammalian Munc 13-1 [6]. A reduction of synapsin I and II was reported in the PD brain at Braak stage I and II [81]. Moreover, various human α-syn transgenic mouse lines [203] and also α-syn oligomers have been found to induce the selective lowering of synapsin I and II, exacerbating memory deficits [204]. Of note, synapsin I was identified among the protein binding to oligomeric α-syn [205]. However, synapsin I and II are not particularly relevant for the modulation of nigrostriatal DA release that is pivotally affected by synapsin III [206,207]. On this line, we described how α-syn can selectively bind and cooperate with synapsin III to modulate DA release from nigrostriatal neurons (Figure 3) [208]. Interestingly, in agreement with evidence showing a marked increase of synapsin III levels in post-mortem PD brains at Braak stage I and II [81], we also found that synapsin III is accumulated in the caudate putamen of PD patients and is associated with α-syn LB insoluble fibrils [80,208]. More recently, we observed that synapsin III knock out (KO) mice do not develop α-syn fibrillary aggregates, synaptic changes and nigrostriatal degeneration following overexpression of human wt α-syn by adeno-associated viral injections [78], thus strongly supporting the idea that synapsin III is a crucial mediator of α-syn aggregation. Recently, Kouroupi and coauthors [209] described a significant reduction of synapsin III in induced pluripotent stem cell (iPSC)-derived neurons from A53T mutant patients, while we did not observe any particular difference in synapsin III expression between iPSC-derived dopaminergic neurons from MSA patients, which only showed a slight and non-significant increase of α-syn levels [210]. These findings support the idea that synapsin III may be differentially affected by/impinge on the distinctive α-syn structural changes occurring in familial PD or in MSA. Synaptic vesicle glycoprotein 2C (SV2C) is another modulator of the vesicular function that is thought to interact with α-syn and affect its aggregation [211]. At the active zone, α-syn is not only responsible for the mobility of SV, but together with Cistein String Protein α (CSPα) (Figure 3) [7,9,212], acts as a chaperone, supporting Soluble NSF Attachment Protein Receptors (SNARE) complex assembly and distribution [213]. The chaperone role of α-syn has been associated with its direct binding to synaptobrevin/VAMP 2 and phospholipids of the SV [213,214,215] (Figure 3). The SNARE complex has been found to be perturbed in PD and DLB patients and in experimental models [78,81,82,83,197,216,217]. Moreover, α-syn affects the rate of neurotransmitter release by accelerating the kinetics of individual exocytotic events, promoting cargo discharge and reducing pore closure [218]. Alpha-synuclein seems also to control SV endocytosis [219,220,221], probably by influencing the curvature of the membranes. Synapses are also the sites where β-syn is most abundant. This protein, likewise γ-syn, has been found to inhibit α-syn aggregation [222,223,224,225]. Alpha-, β- and γ-syn do not interact in their monomeric free state supporting that the mechanism by which β and γ-syn retard α-syn aggregation is mediated by some other effect [226]. Consistently, it has been found that β-syn can inhibit lipid-induced aggregation and secondary nucleation of α-syn by competing for binding sites at the surfaces of lipid vesicles and fibrils [227], a finding that confirms the central role of α-syn/lipid interactions in initiating fibrillation. This notwithstanding, more recent data support a weak and transient binding interaction in the micromolar range for α-syn/β-syn [47,228]. In particular, that C-terminal acidic residues of β-syn interact with a “hot spot” region in the N-terminal portion α-syn comprising residues 38−45 and prevent fibrillation of α-syn [228]. Given the high sequence homology between α- and γ-syn at the N-terminus [226,228], it may be feasible that β- and γ-syn interaction may have similar features, while α-syn/γ-syn binding may occur through different modalities that may involve their intrinsically disordered regions [228]. Besides the trafficking of SV, α-syn can also regulate the rate of neurotransmitter release, and of DA in particular. Indeed, α-syn interacts with and affects vesicular monoamine transporter 2 (VMAT2) that is responsible for SV DA uptake (Figure 3) [229,230]. Knock out of α-syn increases VMAT2 expression [229], while α-syn overexpression inhibits VMAT2 activity. VMAT2 distribution and levels are perturbed in experimental models of synucleinopathies [78,231] and VMAT2 is a LB component [232]. Rabs, which, aside from modulating axonal trafficking, are also very important for the regulation of every single step that leads to the release of SV trafficking, docking and fusion at synaptic sites [233], have also been found to interact with α-syn. The protein actively interacts with and is also influenced by numerous members of this superfamily of proteins, such as Rab3a, Rab4, Rab5, Rab7, Rab8a, and Rab11 [194,195,234,235,236,237,238,239] (Figure 2B). Rab3a is involved with α-syn in the tethering of the SV at the synaptic membranes and its homeostasis is disrupted by α-syn overexpression [235,237]. This phenomenon also impairs the ER-Golgi trafficking [237]. Rab5 is involved in keeping the size of SV uniform by preventing their homotypic fusion [240]. The overexpression and aggregation of α-syn induces a redistribution of Rab3a, Rab5 and Rab11 [78,195]. This impairment of Rabs organization can block the clearance of misfolded proteins and enhances the spreading and uptake of α-syn in parallel. Rab4, Rab5 and Rab7 resulted as crucial mediators for the induction of this vicious circle [194,236,239], together with phospholipase D1 [241]. This evidence supports the idea that the chimeric behavior of α-syn at synaptic terminals can induce a multi-faceted misregulation of many other synaptic proteins that further contributes to propagate synaptic damage with a boosting-like fashion.

## 5. Alpha-Synuclein Modulation of Neurotransmitter Reuptake and Receptors

Dopamine signaling in presynaptic terminals is strictly controlled by the DA transporter (DAT) that governs the reuptake of DA and regulates dopaminergic neurotransmission. The regulation of DA uptake is controlled through the redistribution or the internalization of the DAT that can be modulated by kinases, such as PKC or PKA [242]. Indeed, PKC activation can decrease DAT function by inducing its sequestering [243]. Dopamine transporter internalization can occur through a constitutive endocytic pathway or via PKC-dependent endocytosis that is mediated by clathrin [244] through the formation of early endosome that interacts with Rab5 [245,246]. Moreover, DAT internalization has also been found to be dynamin-dependent [247]. Dopamine transporter functions are also controlled by α-syn, as mice not expressing α-syn show impairment of DAT functions and reduction of striatal DAT levels [15,248]. A direct interaction between α-syn and DAT has been reported in PD patients as well as in experimental models [15,79,249,250,251] (Figure 2B), where it is believed that α-syn controls the trafficking of DAT by modulating the cytoskeleton and DAT anchoring. In physiological conditions, α-syn binds the C-terminal tail of DAT, increasing its levels at the plasma membrane with a consequent enhanced uptake of extracellular DA [252]. Mutant α-syn decreases the trafficking of DAT at the plasma membrane [253] with a clathrin-dependent mechanism [244,254]. However, enhancement of DAT activity increases the levels of DA, resulting in production of ROS, which is also mediated by the phosphorylation of α-syn [255]. Similarly, α-syn controls the trafficking of serotonin and norepinephrine through their transporters [256]. Notably, post-mortem analysis of the brains of patients affected by PD showed a significant decrease of the levels of serotonin transporter (SERT) and norepinephrine transporter (NET) [256,257]. The monoaminergic transporters are modulated by the interaction of the C-terminal domain of α-syn that removes the transporters from the plasma membrane and increases their compartmentalization with the contribution of the microtubule networks [258,259,260,261]. According to this, α-syn mutations affect serotoninergic fibers by decreasing their density and serotonin levels [262]. The presence of α-syn fibrils changes the functional interactions of native α-syn not only regarding NET but also SERT, thus perturbing the re-uptake of the transporters [263]. Moreover, the C-terminal region of aggregated α-syn interacts with serotonin stabilizing the α-syn oligomers and blocking the formation of α-syn fibrils [264]. Dopaminergic neurotransmission is also controlled by DA D2 and D3 receptor (D2R, D3R). Alpha-synuclein enhances the DA-mediated intracellular signaling pathways by D2R [265], while the treatment with D2R/D3R agonists is able to counteract α-syn aggregation [249,266]. Other putative receptors have been reported to interact with α-syn at the synapse, such as the cellular prion protein (PrPC) [267], neurexin [268], amyloid β precursor-like protein 1 (APLP1) and lymphocyte-activation gene 3 (LAG3) [269]. These receptors are thought to be involved in α-syn pathological actions by mediating the binding and internalization of α-syn fibrils, contributing to their cell-to-cell transmission.

## 6. Concluding Remarks

Although this review only describes the synaptic components of α-syn interactome, the overview is highly representative of its multifaceted structural flexibility and neurophysiological complexity. Alpha-synuclein is cardinal in orchestrating the synaptic machinery. The huge number of protein partners that it influences, or with which it interacts, coupled with the fact that α-syn can easily shift in conformation, render it difficult to disclose what its most relevant action is. Consequently, it is still challenging to imagine what may be the most dramatic molecular consequences of α-syn aggregation at synaptic sites in the very early phases of synucleinopathies. Another key relevant aspect to be considered following the multifaceted neurobiology of α-syn described above is the possible impact of therapeutic strategies aimed at reducing pathological α-syn burden in synucleinopathies. While it is foreseeable that agents reducing α-syn fibrillation or promoting its clearing may be beneficial, strategies aimed at an overall reduction of α-syn may entail a complete loss of the regulatory actions of the protein at synaptic and extrasynaptic sites, thus further compromising neuronal homeostasis and function. This notwithstanding, some key α-syn synaptic interactants are emerging as new participants in the induction of α-syn-aggregation-related synaptic damage, thus offering a more comprehensive overview of the pathophysiological signature of synucleinopathies and new hopes for therapeutic development. Further studies are needed to disclose whether α-syn partners may be crucial in the onset of synucleinopathies and may constitute novel therapeutic targets for their cure.
